# Prevalence and Data-Driven Exploration of Pre-Diagnostic Symptoms and Features of Gilbert’s Syndrome in the UK Primary Care Population

**DOI:** 10.2147/CLEP.S520589

**Published:** 2025-08-28

**Authors:** Rini S S Veeravalli, Laura J Horsfall, Kenan Direk, Irene Petersen

**Affiliations:** 1 Research Department of Primary Care and Population Health, Institute of Epidemiology and Health Care, University College London, London, UK; 2 Imperial Clinical Trials Unit, School of Public Health, Imperial College London, London, UK

**Keywords:** Gilbert’s syndrome, hyperbilirubinemia, symptomology, electronic health records, machine learning, diagnostic triggers

## Abstract

**Background:**

Gilbert’s syndrome (GS) is a common genetic disorder marked by elevated bilirubin levels due to UGT1A1 enzyme deficiency. While jaundice and some adverse drug reactions are the primary recognised clinical features, individuals with GS frequently report non-specific symptoms like fatigue, brain fog, and abdominal pain. This study investigates the symptoms and diagnostic triggers of GS using UK primary care electronic health records.

**Methods:**

We analysed data from the IQVIA Medical Research Database, covering over 11 million active UK patients. Individuals with a recorded GS diagnosis were identified and their sociodemographic profiles described. Using a nested case-control design, we applied machine learning-based feature selection to pinpoint key clinical features recorded up to five years before diagnosis. These features were then examined longitudinally by sex to distinguish persistent symptoms from short-term diagnostic triggers.

**Results:**

The estimated UK prevalence of GS was 180.4 per 100,000 (95% CI: 174.4–186.6), with diagnoses more common in men, peaking around age 35, and more frequent in areas of least social deprivation. Among 9,240 GS cases and 150,846 controls, machine learning identified key diagnostic themes including jaundice, abnormal liver function tests, abdominal pain, fatigue, bowel changes, and sleep disturbances. While most of these features appeared primarily in the year prior to diagnosis, only abdominal pain and fatigue were consistently more common in GS cases up to five years before diagnosis.

**Conclusion:**

Our findings highlight both expected and novel GS diagnostic triggers. While many features likely reflect known symptomology or incidental detection via routine testing, the persistent presence of fatigue and abdominal pain suggests they may be under-recognised symptoms of GS. These findings warrant further investigation, and the data-driven approach used here may help uncover early signs of other underdiagnosed genetic conditions.

## Plain Language Summary

Gilbert’s syndrome is a common genetic condition causing higher levels of bilirubin in the blood due to a liver enzyme deficiency, leading to jaundice and certain adverse drug reactions. Many people with this syndrome also report other symptoms like fatigue, brain fog, and stomach pain. This study used health records from over 1.8 million UK patients to explore these symptoms and diagnostic triggers. While jaundice was a key diagnostic trigger, abdominal pain and fatigue were more common in people with Gilbert’s syndrome compared to control patients up to five years before diagnosis. The findings suggest that using large-scale health data could help us to better understand symptoms of common or rare genetic conditions that have a delayed diagnosis.

## Introduction

Gilbert’s syndrome (GS) is a genetic liver disorder characterized by the body’s reduced ability to process bilirubin, a byproduct of red blood cell catabolism. The underlying causes in most human populations studied are functional variants in the regulatory and coding regions of the UDP glucuronosyltransferase family 1 member A1 (*UGT1A1)* gene that reduce activity by 30–50%. UGT1A1 is the sole enzyme responsible for converting insoluble neurotoxic (unconjugated) bilirubin to soluble (conjugated) form for elimination, although variants in other enzymes may play a role in determining serum bilirubin levels.^1^,^2^ GS is typically diagnosed by exclusion through blood tests that detect elevated levels of unconjugated bilirubin, without haemolysis or structural liver damage.^3^ GS may become apparent after birth or in late teens and more commonly diagnosed in males than females.^4^ Known diagnostic triggers include infections, low-calorie diets, pregnancy, surgery and extreme exercise.^5^,^6^ However, diagnosis can often occur incidentally during unrelated medical visits that require blood testing. Genetic testing can be performed to confirm diagnosis.^7^

Typically, GS is considered to be clinically benign, with episodes of mild jaundice as the main recognised symptom (yellowing of skin, mucous membranes, and whites of eyes). However, for some people with GS, these episodes of jaundice can be frequent and prolonged (>19 days) leading to a reduced health-related quality of life.^6^ Recent investigations have expanded symptomatology, particularly regarding gallstone disease, dyspepsia (abdominal discomfort due to indigestion), and loss of appetite, in specific populations with a small to moderate number of clinically and genetically diagnosed individuals.^6^,^8^ The UGT1A1 enzyme is also responsible for eliminating certain drugs, and people with GS may be susceptible to serious adverse effects.^4^,^9^,^10^ For example, routine screening for *UGT1A1* variants underlying GS is recommended in the United States prior to irinotecan chemotherapy to identify patients at risk of life-threatening toxicity.^9^ Furthermore, people with GS often co-inherit functional variants in other UGT1A isoforms expressed in the gastrointestinal system that are responsible for processing other exogenous and endogenous substances such as hormones.^11^,^12^

Large-scale explorative studies covering a range of GS symptoms in a broadly representative population of diagnosed individuals have been lacking. Such research could aid in earlier recognition of the condition, potentially reassuring patients with unexplained symptoms and optimising drug monitoring, especially for medications reliant on UGT1A1 for elimination. Recent studies in medical literature that aimed to predict disease-related events and/or reduce diagnostic time found use of machine learning feature selection techniques to improve classification performance.^13–15^ These approaches that recognise patterns from extensive patient data may be useful for symptom discovery, especially for underdiagnosed conditions regarded as asymptomatic, such as GS, where genetic testing is not routinely available.

The present study has two aims. First, to describe the sociodemographic factors associated with a GS diagnosis in UK using a large representative sample of electronic primary health care records. Second, to identify and differentiate sex-specific diagnostic triggers and symptoms of a GS diagnosis using a machine learning approach combined with a subsequent longitudinal analysis.

## Materials and Methods

### Study Design and Participants

We used the IQVIA Medical Research Database (IMRD), one of the UK’s largest longitudinal primary health care datasets. In the UK, primary care is the first point-of-contact for patients, and includes general practice (GP), pharmacy, and eye health, midwifery, and dental services. Like all public health care, it is offered for free by the National Health Service (NHS) to all “ordinarily resident” in the UK. Most of the UK population are registered with a GP, which records encoded and free-text patient and medical information collected during consultations as well as co-ordinate patient care across other primary and secondary care settings.^16^ The IMRD encompasses patient demographics, prescriptions, and symptoms and disease diagnosis recorded using Read codes for over 18 million patients across 797 practices. It contains anonymised electronic records derived from staff consultations at each practice and includes data from The Health Improvement Network (THIN) database, owned by Cegedim, which IQVIA accessed via a sublicense.

#### Recording of Diagnoses and Symptoms

The Read code system is extensively used in primary care settings across the UK, enabling general practitioners to record patient encounters, diagnoses, and treatments efficiently.^17^ Read codes are structured hierarchically; read left to right, they become increasingly specific with each subsequent character.^18^ We used the latest and last Read code Version 3, which contains over 100,000 Read codes consisting of 7 characters. Cases were identified as individuals with the GS diagnostic Read code, “C374200”, using the method to create medical code lists for primary care case identification described elsewhere.^19^

The reasons for a patient receiving this diagnosis during consultation would be decided by the individual GP and is expected to be based on UK national diagnostic guidelines from the National Institute for Health Care and Excellence (NICE) as outlined in the introduction, which state typical raised serum bilirubin concentrations would not exceed 68–85 micromol/L and that normally total bilirubin is less than 17 micromol/L.^3^ Bilirubin fractions or other potential factors that can increase total bilirubin are not always recorded in primary care. Hence, we do not explicitly use or evaluate patient serum bilirubin level or genetic confirmation, as GS genetic testing is uncommon in the UK, in this study to identify cases. Having previously investigated bilirubin levels for people diagnosed with GS in this data source, the objective of this study is to understand which GP recorded events precede the diagnostic Read code.^20^

### Sociodemographic Patterns: Cohort 1

First, we aimed to establish how common GS is recorded in the primary care setting and describe prevalence by sociodemographic factors. Data from 1^st^ January 1998 to 31^st^ December 2018 were included for individuals currently alive and/or permanently registered at a practice contributing data to the database as of 16th January 2019. Each eligible individual entered the study at their registration date, post their practice’s acceptable mortality rate (AMR) and acceptable computer usage (ACU) dates, ensuring availability and acceptable quality of their full medical records.^21^ Exclusions were made for individuals with missing age and sex values, as well medical record events with missing medical codes or dates. Social deprivation was represented by a Townsend score, ie, quintiles of the Townsend deprivation index, which denotes 1 as least deprived and 5 as most deprived residential areas. Townsend scores closest to registration date were included for individuals with social deprivation information.^22^ Eligible individuals, born in any year, were followed-up from registration until the earliest of the study end date (31st December 2018), their practice’s last date of data, their transfer date, and their death date, and this time period is defined as follow-up length (Figure S1).

Cases could have a diagnosis at any point in their medical records before the study end date. Cases with multiple diagnosis events were assigned the earliest recorded GS diagnosis date.

### Diagnostic Triggers and Symptoms: Cohort 2

For the study’s second objective on diagnostic triggers and symptoms, a case–control sample was created following the same inclusion criteria as the prevalence cohort with some adjustments: individuals exited the study at the earliest of their practice’s last date, transfer date, death date, the study end date (31st December 2018), or their 100^th^ birthday. Patients registered for less than one year and cases lacking at least one year’s worth of data before diagnosis were excluded to ensure adequate follow-up. Cases were further excluded if their first diagnosis date fell outside the study period.

Cases were again identified using the GS diagnostic Read code, “C374200”, and were matched to controls in a 1:20 ratio by age (year of birth), sex, GP practice, and diagnosis date using an incidence density sampling based method, matching without replacement.^23^ Controls were assigned the diagnosis date of their matched case (index date), ensuring that the date fell within the control’s follow-up period and occurred at least a year after their start date.

Electronic health care records (EHRs) from cases and matched controls within the five-year period leading up to the first diagnosis were collected, excluding records with missing event dates. To perform feature selection, records underwent transformation: i) retaining only the first occurrence of a medical code if multiple were present, ii) converting from long to binary wide format suitable for machine learning analysis. In this format, each row represents a patient, and each binary column indicates the presence or absence of a feature (a Read code), ensuring the independence assumption of regression by removing duplicate measurements.^24^ Subsequently, features not part of a curated set of relevant phenotypic features were excluded (see Supplementary). The sample was then stratified by sex.

To ensure high quality and non-prevalent symptom reporting in estimating feature incidence in the five years prior to diagnosis, each patient’s start time was updated and defined as the latest date among: six months after registration, the practice’s AMR and ACU dates, and 1–6 years before diagnosis.^25^ Individuals (cases and matched controls) with less than 6 months of records between the new start date and diagnosis (recorded or assigned) were excluded. To ensure child patient data were included, individuals were not required to have at least five years of records. See Figure S2 that illustrates sample selection for an example individual.

### Statistical Analysis

#### Cohort 1

GS period prevalence was estimated in Cohort 1 as the number of cases per 100,000 people between 1998 and 2018, inclusive, and stratified by age, sex, year, time in study, and social deprivation score. All rates were estimated with 95% Confidence Intervals assuming a Poisson distribution.

Age at first diagnosis was estimated in all 21,174 GS cases identified in the IMRD, stratified by sex and whether the first diagnosis occurred within the first year of GP registration, to account for potential misclassifications of past diagnoses as new incidents when patients switch GP practices.^25^

#### Cohort 2

Cross-validated Recursive Feature Elimination was used for feature selection to output a subset of the most important pathological and phenotypic Read code features (inputted at a four-character level, to balance complexity and descriptive power) recorded in the five years before GS diagnosis (see Supplementary for details). Importance was quantified using Random Forest Permutation Importance (RF-PI) with five-fold cross-validation and five permutations per fold, where the higher a feature’s PI value, the more important that feature is in enabling the model to correctly distinguish between cases and controls. Default hyperparameter values were used, except for the number of RF trees to build (n = 1,000), to maintain simplicity to allow replicability. Resulting important features were then investigated across both sexes, each defined with seven-character Read code lists which were created using the method mentioned previously.

Symptom frequency was determined and a chi-square test assessed differences in symptom occurrence between cases and controls in the matched sample.^26^ Symptom incidence, for each year in the 0–5 years prior to diagnosis, was estimated for cases and controls and presented as annual incidence rates (per person-year) with 95% confidence intervals assuming a Poisson distribution for each symptom.^27^ Incidence was calculated by dividing the total new symptom events by the sum of person-years contributed by all individuals in the sample for each year. Results were stratified by sex to observe differences in men and women. Time from first symptom presentation to first diagnosis of GS was estimated for all cases in the matched sample.

Analysis was conducted using Stata MP version 17, Python version 3.6 and 3.9.7.^28^,^29^,^29^,^30^

## Results

### Sociodemographic Patterns: Cohort 1

The cohort comprised 1,899,529 individuals with a median follow-up time of 12 (IQR 6–17) years (Table 1). In total, 3,424 GS cases were identified, giving a clinically recognised prevalence of 180.4 per 100,000 people (95% CI: 174.4–186.6). The highest GS prevalence was observed in the 15+ years age band: 201.7 (194.8–208.7) per 100,000 people. Prevalence was nearly twice as high in men (237.4 (227.7–247.5)) than women (124.5 (117.5–131.8)) per 100,000 people. Median age at first recorded diagnosis was 35 years (IQR 21–53) for men and 33 years (IQR 22–47) for women. Recorded diagnosis was almost twice as high in the least deprived Townsend quintile compared to those with the most deprived Townsend quintile (Table 2). See Figure S3 for histograms estimating age at first diagnosis.

**Table 1:** Summary of Cohort 1 Characteristics Before Prevalence Analysis*

Population size, N	1,899,529
Male, N (%)	944,337 (49.71)
Female, N (%)	944,337 (50.29)
Median follow-up length, years (IQR)	12.00 (6.31–16.91)

**Notes**: *During estimation of prevalence, exclusions of diagnosis events before each participant’s start and/or after end date may exclude participants from the cohort.

**Table 2:** Prevalence Estimates for Gilbert’s Syndrome. Prevalence Rates are per 100,000 People

	GS Diagnosis Per 100,000 People
	(95% CI)
Period prevalence	
1998–2018	180.4 (174.4–186.6)
Sex	
Male	237.4 (227.7–247.5)
Female	124.5 (117.5–131.8)
Age (years)	
0–4	1.2 (0.3–3.1)
5–9	2.9 (1.4–5.2)
10–14	39.4 (33.1–46.6)
15+	201.7 (194.8–208.7)
Time in study (years)	
0–4	66.5 (62.9–70.4)
5–9	62.4 (58.5–66.4)
10–14	70.7 (65.7–75.9)
15+	66.3 (60.3–72.8)
Year*	
1998–1999	11.8 (7.5–17.5)
2000–2009	92.5 (87.0–98.3)
2010–2014	66.1 (62.3–70.1)
2015–2018	64.9 (61.3–68.6)
Townsend score quintile	
(Least Deprived) 1	226.4 (211.9–241.6)
2	198.8 (185.3–213.1)
3	182.6 (170.0–195.9)
4	155.6 (143.2–168.7)
(Most Deprived) 5	120.3 (108.0–133.6)

**Notes**: *These intervals were chosen to ensure there were enough cases per interval for the results to be shown in accordance with the data licence.

### Diagnostic Triggers and Symptoms: Cohort 2

The case sample included 6,152 (67%) males and 3,088 (33%) females, reflecting known GS prevalence by sex (Table 3).^4^ Feature selection across the matched cohort identified the following Read code themes associated with GS: jaundice, disorders of bilirubin metabolism (unspecified), abnormal liver function test, abdominal pain, nausea, fatigue, eyes (swollen, dry, red), urine (altered control), change in bowel movement, swallowing, sleep, depression. In both men and women, Read code features relating to jaundice were consistently found to be most important for determining GS case status in the five years prior to diagnosis (in decreasing order of importance): 1675 yellow/jaundiced colour, R024 jaundice (not of newborn), 14C6 history of (H/O) jaundice, and C37X disorder of bilirubin metabolism, unspecified.

**Table 3:** Summary of Cohort 2 Characteristics

	Total (with Any Follow-Back Data)	
	Case (n=9,240)	Control (n=150,846)
Male, N (%)	6,152 (67%)	96,980 (64%)
Female, N (%)	3,088 (33%)	53,866 (36%)
Median age at diagnosis, years (IQR)	44 (29–60)	44 (29–60)
Median record length before diagnosis, years (IQR)	6 (3–11)	7 (4–11)

Table S1 displays the Read code lists created to identify patients with the symptoms identified by feature selection, and Table 4 displays the cross-sectional recording of these symptoms within five years of the index date for cases and controls. Overall, features such as abdominal pain, fatigue, jaundice, changes in bowel habits, bilirubin disorders and abnormal liver function tests were more frequent in cases than controls (p<0.00001). Jaundice appears in only 3% of cases and in less than 1% of controls. Depression was slightly lower in cases versus controls (8% vs 9%, p=0.00013). There was no association with swallowing difficulties or sleep disturbances. Disorders of bilirubin metabolism (unspecified), abnormal liver function test, and swallowing difficulties were 1% or less in all groups of individuals and were excluded from the longitudinal analyses due to low sample size.

**Table 4:** Frequency of Symptoms in People with Clinically Recognised Gilbert’s Syndrome and Frequency-Matched Control Sample

Symptom	Male		Female		All (Males and Females)		P-value**
	Case (n=6,152)	Control (n=96,980)	Case (n=3,088)	Control (n=53,866)	Case (n=9,240)	Control (n=150,846)	
Abdominal pain	1151 (19%)	11,196 (12%)	784 (25%)	10,006 (19%)	1935 (21%)	21,203 (14%)	< 0.00001
Fatigue	724 (12%)	5798 (6%)	677 (22%)	6780 (13%)	1401 (15%)	12,581 (8%)	< 0.00001
Eyes (swollen, dry, red, infected eyes and eyelid)	586 (10%)	8560 (9%)	387 (13%)	6286 (12%)	973 (11%)	14,845 (10%)	0.031
Depression	388 (6%)	6612 (7%)	367 (12%)	7520 (14%)	755 (8%)	14,122 (9%)	0.00013
Nausea	321 (5%)	3724 (4%)	336 (11%)	4209 (8%)	657 (7%)	7934 (5%)	< 0.00001
Urine (altered control)	336 (4%)	4166 (4%)	207 (7%)	2927 (5%)	543 (6%)	7089 (5%)	< 0.00001
Sleep disturbances	193 (3%)	3296 (3%)	129 (4%)	2165 (4%)	322 (3%)	5461 (4%)	0.50
Jaundice	233 (4%)	108 (<1%)	89 (3%)	59 (<1%)	322 (3%)	166 (<1%)	< 0.00001
Change in bowel habits	123 (2%)	1069 (1%)	60 (2%)	676 (1%)	183 (2%)	1742 (1%)	< 0.00001
Swallowing difficulties	54 (<1%)	784 (<1%)	38 (1%)	417 (<1%)	92 (1%)	1202 (<1%)	0.38
Abnormal liver function test	77 (1%)	170 (<1%)	15 (<1%)	78 (<1%)	92 (1%)	250 (<1%)	< 0.00001
Disorders of bilirubin metabolism (unspecified)	38 (<1%)	- (<1%)*	13 (<1%)	- (<1%)*	51 (<1%)	- (<1%)*	< 0.00001

**Notes**: *Counts are omitted if representing groups of less than 7 individuals (denoted with “-”). Symptoms are ordered by frequency. The last 3 conditions (shaded grey) were not included in further analysis. **Chi-square test.

The longitudinal analysis was used to look retrospectively at a five-year period before diagnosis and examine whether symptoms are persistently higher in GS cases versus their matched controls or only emerge close to the diagnosis date. All features increased in the year before GS diagnosis (Figure 1). Incidence rates for jaundice (the least common feature analysed) were slightly higher in cases between 3 and 5 years before diagnosis. Rates diverge gradually at 1–3 years before diagnosis when incidence in cases increased compared to controls. There was a higher incidence of eye (redness, dryness, infection, eyelid infection) symptoms in cases compared to controls (but estimates were imprecise due to low numbers).
**Figure 1:**
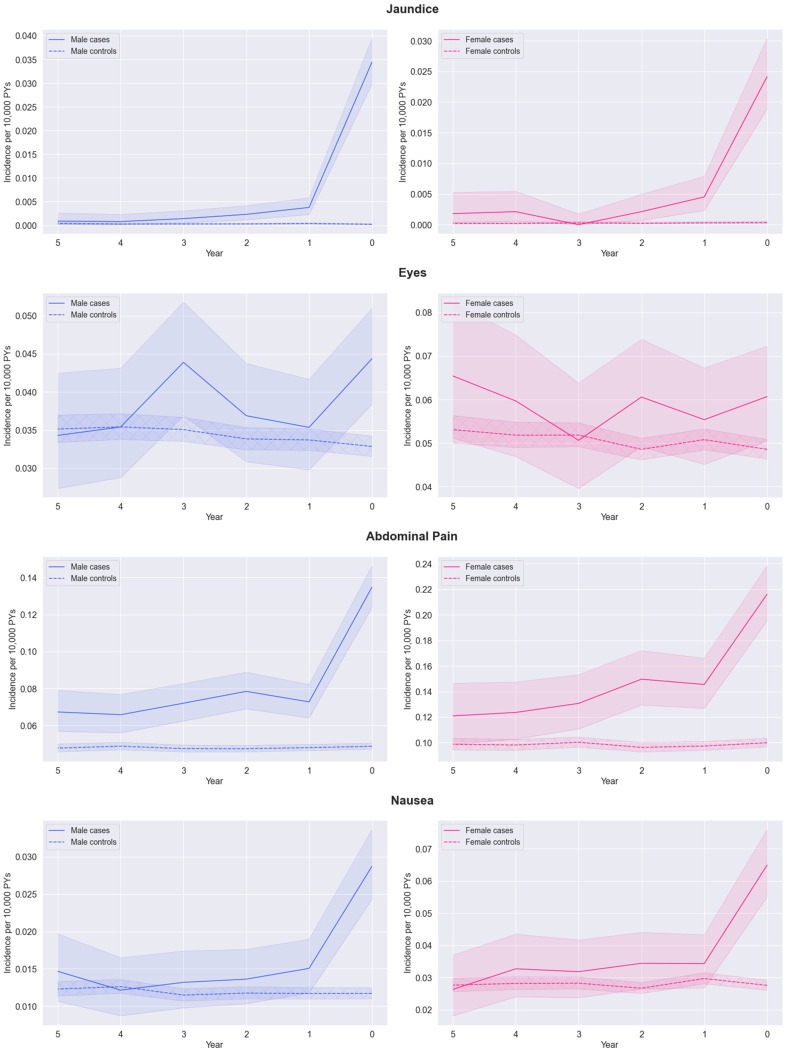
Continued.

**Figure 1:**
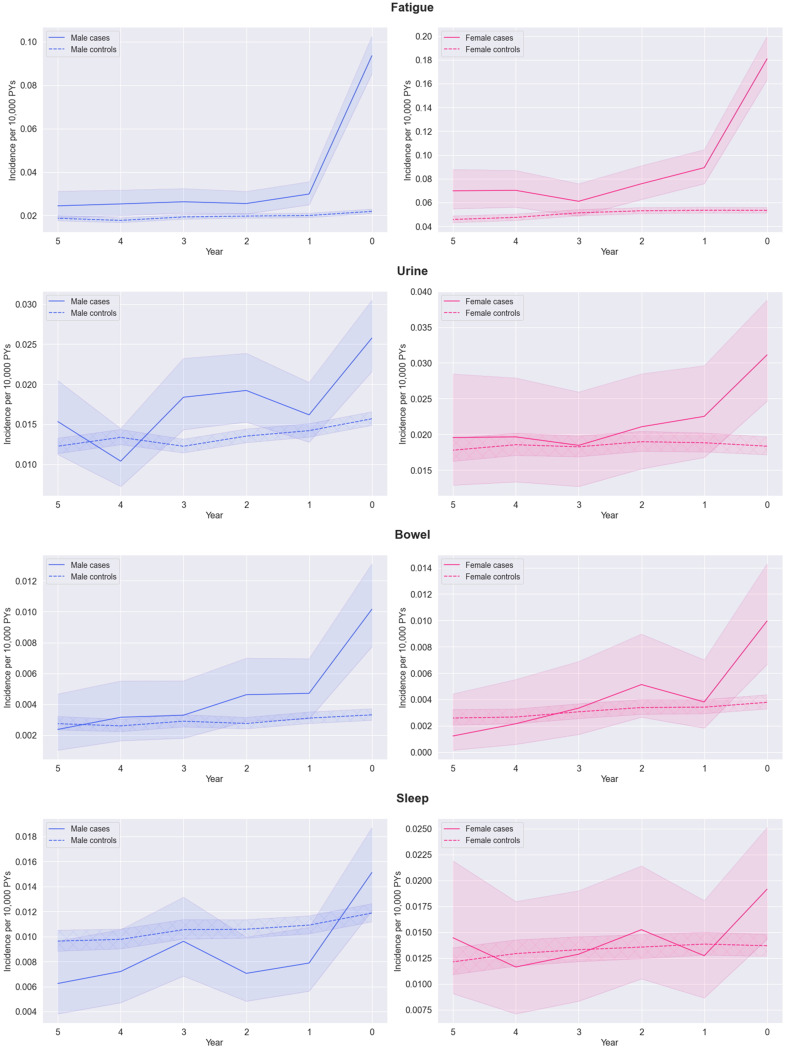
Continued.

**Figure 1:**
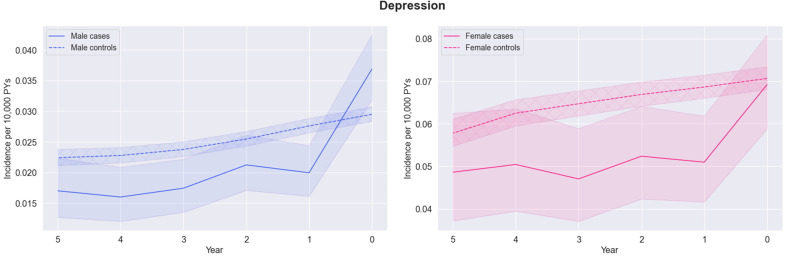
Earliest incidence of jaundice*, eyes (swollen, dry, red), abdominal pain, nausea, fatigue, urine (altered control), change in bowel habits, sleep (difficulties), and depression symptoms (per 10,000 Person-Years, with 95% confidence intervals) at each year in the five-year period before Gilbert’s syndrome diagnosis split by case status for i) men (left) and ii) and women separately (right). *The 95% confidence intervals for jaundice symptoms are very small due to infrequency of these symptoms.

Nausea, sleep disturbance and urine or bowel changes were broadly similar in cases and controls in time periods earlier than one year before the index date. However, abdominal pain and fatigue remain higher in GS cases than controls up to five years before the index date. Depression remained lower in GS cases than controls up to five years before the index date.

Except for jaundice, most features were more common in women, but sex did not appear to be a strong modifier in the relative trends in features leading up to the index date.

## Discussion

### Summary

We have shown that, relative to population estimates of between 2% and 10%, GS is rarely diagnosed in the UK primary care setting at just 0.18% of patients.^31–33^ Around half of people receive a first recorded diagnosis after the age of 34–35, and these tend to be men, and people living in the least deprived areas. Feature selection identified codes relating to bilirubin and jaundice as well as some non-specific symptoms such as nausea, fatigue, depression and abdominal pain. Most symptoms were higher in GS cases compared with controls except for depression, which was lower. By examining the first consultation of these symptoms up to five years before the index date, we found that fatigue and abdominal pain were the only features that were consistently higher in GS cases than controls during this period. Most other non-specific features were only present in the year before GS diagnosis and are likely triggers rather than true symptoms primarily caused by a UGT1A1 enzyme deficiency.

### Comparison with Other Studies

The observed prevalence is lower than population estimates, but it is expected that prevalence in clinical databases will be lower than in the community as a number of people are undiagnosed or do not have a record in EHR, and because we only have data for the time that patients are registered with their GP rather than lifetime follow-up. GS is often clinically diagnosed incidentally and is considered benign; therefore, GPs may see little value in spending resources to make and record a diagnosis. GS prevalence is known to differ by population and vary by ethnicity, and GS is caused by different genetic variants of the *UGT1A1* gene in different populations.^34–39^ Studies using genetic diagnosis rather than clinical diagnosis found prevalence is higher, which suggests that penetrance is low, ie, a larger proportion of people have the genetic makeup for GS than present symptoms that lead to diagnosis.^40–42^ Diseases with low penetrance will likely be underestimated if identified by clinical codes. For instance, the most similar and recent European study looking at associations between GS genetic confirmation and clinical diagnoses found only 3% of genetically confirmed European cases in the UK Biobank had a coded EHR diagnosis, however this population is not representative of the UK. Additionally, GS diagnostic thresholds for elevated serum bilirubin levels may vary between populations and studies, contributing to differences in reported prevalence.^43^ Where other GS studies, including the aforementioned UK Biobank study, look for outcomes cross-sectionally once diagnosis is known, our study also looks at non-specific symptoms longitudinally in the time leading to diagnosis in the largest sample to date.

The finding that almost twice as many men (0.24%) than women (0.12%) are diagnosed with GS is consistent with findings in literature of the high male-to-female ratio.^32^,^33^ This reflects the higher mean bilirubin levels in men and that women are less likely to meet the typical bilirubin threshold for diagnosis.

We found evidence suggesting abdominal pain and fatigue might be persistent symptoms of GS rather than simply diagnostic triggers. A longitudinal study of 100 GS adults identified fatigue and abdominal pain as substantially higher than in controls (p<0.0001) but the method of recruitment could have led to information bias.^6^ While robust recent studies on symptoms of GS to support our findings are lacking, there is a biological plausibility to higher rates of abdominal pain and fatigue. For example, red blood cell lifespan in people with GS is reported as 30% below undiagnosed controls and this mild haemolysis could cause symptoms of fatigue.^44^,^45^ There is also evidence from genetic studies that UGT1A1 deficiency can increase the risk of gallstones, which could increase primary care consultations for abdominal pain.^2^,^42^ Establishing whether abdominal pain is a true symptom is important because in the UK the NICE diagnostic guidelines for GS suggest considering alternative diagnoses if symptoms of abdominal pain are present.^3^

## Strengths and Limitations

This study is the largest and most recent study of features and potential symptoms presenting before a GS diagnosis. The data analysed are representative of the UK population and clinical practice, and therefore results can be extrapolated to refer to the wider UK population.^46^ The large sample size allowed to investigate trends of symptom occurrence over five years and monitor time from symptom occurrence to first diagnosis in a longer period using a contemporary population (with data collected up to the start of 2019). The longitudinal study design minimises recall bias, unlike similar studies that rely on retrospective self-reporting of symptoms, as using routinely collected EHRs reduces the risk of misclassification or event omission and improves data reliability and validity. Use of machine learning (ML) and feature selection techniques that correctly identified important features of GS acts as a proof of concept for similar methods to be meaningfully applied to understanding the symptomology of other diseases in future studies without requiring complex knowledge or resources.

Future studies with increased computational power could use methods that account for correlation between features, which can influence ML feature importance values. Multiple consultations with the same symptom could reveal more information if kept in analysis, and analysis of co-occurrence of symptoms in relation to other symptoms, such as jaundice, may be useful. Further limitations include low sample sizes and selection bias. Even with a large dataset such as IMRD, we may have lacked power to investigate rare features. Depression was the only symptom that was clearly higher in controls than in cases (except in the year before diagnosis). This is probably due to a higher GS diagnosis rate in areas with less social deprivation (Townsend quintile 1) where people have lower rates of depression.^47^ Differences in social deprivation are unlikely to explain the higher rates of fatigue in GS as primary care consultations with fatigue symptoms are also more common in the most socially deprived areas.^48^ Primary care consultations with chronic pain are also more common in socially deprived areas, although data specifically for abdominal pain by social deprivation are lacking.^49^ In this analysis, we used the date that a diagnostic code for GS was entered into the patient record. It is plausible some of the cases were aware of their diagnosis before GP recorded diagnosis, and this might have biased the results. Additionally, since we had access to a clinical database rather than a research database, use of clinical diagnosis without genetic confirmation may introduce misclassification, although it is very likely that those with a diagnosis are true cases due to our very specific case finding approach. Future studies may benefit from the analysis of ethnicity data as GS prevalence is known to vary between populations. This was an exploratory and descriptive study, and future work could approach questions around true symptoms within a causal framework and adjust for potential confounders such as ethnicity and social deprivation.

## Conclusion

### Key Findings

#### Impact

Gilbert’s syndrome is one of the most prevalent but least understood genetic conditions in terms of clinical recognition and broader symptoms. The impact of this study is to better understand the condition by conducting one of the largest studies on features and symptoms presenting in the primary care setting to date. We found that GS is underdiagnosed or diagnosed in adulthood for most patients. By using longitudinal electronic health records, we could attempt to differentiate diagnostic triggers from potential non-specific symptoms. For example, nausea appears to be a diagnostic trigger that is only higher in GS cases than in controls up to a year before diagnosis, whereas abdominal pain and fatigue are more frequent than in controls up to five years before a diagnosis. Future work should examine causal relationships with these non-specific symptoms presenting to primary health care providers, results of which may impact future diagnostic guidelines and clinical decision-making. Advanced methods of risk modelling incorporating multiple consultations and symptom clusters could help identify potential GS cases for confirmatory genetic testing at an earlier age. In practice, this could not only reduce health care resources used to exclude serious liver disease but also identify patients that may have a reduced capacity to process commonly prescribed drugs, preventing potentially serious adverse reactions as well as reoccurrences of jaundice. As with all genetic diseases, quicker identification and management of the symptoms would also improve both the physical and mental health and wellbeing of patients with GS.

## Highlights

- In the UK primary health care setting, most people with recognised Gilbert’s syndrome (GS) are diagnosed after 34 years of age or never diagnosed.
- Clinical recognition of GS was higher for men and people living in the least socially deprived areas.
- Machine learning methods applied identified several features associated with a diagnosis. These included jaundice, disorders of bilirubin metabolism (unspecified), abnormal liver function test, abdominal pain, nausea, fatigue, eyes (swollen, dry, red), urine (altered control), change in bowel movement, swallowing difficulties, sleep disturbances and depression.
- All features were recorded at high levels in the year before diagnosis and appear to be diagnostic triggers.
- Only fatigue and abdominal pain remained higher in GS cases versus controls up to five years before diagnosis but might still represent true symptoms of the condition.

## Impact and Implications

This is the largest study into Gilbert’s syndrome (GS) symptoms in a European setting, using real-world data from over 9,000 cases identified in a representative UK dataset. We found evidence supporting fatigue and abdominal pain as biologically plausible symptoms that require further investigation in terms of causality and impact on quality of life for people with GS.

Relatively simple machine learning methods could be used to identify features of other genetic disorders, which could help characterise symptomology and reduce diagnostic delays.

## References

[1] Bai J, Luo L, Liu S, et al. Combined effects of UGT1A1 and SLCO1B1 variants on Chinese adult mild unconjugated hyperbilirubinemia. *Front Genet*. 2019;10. doi:10.3389/fgene.2019.01073 PMC6834774 31737051

[2] Buch S, Schafmayer C, Völzke H, et al. Loci from a genome-wide analysis of bilirubin levels are associated with gallstone risk and composition. *Gastroenterology*. 2010;139(6):1942–1951.e2. doi:10.1053/j.gastro.2010.09.003 20837016

[3] NICE Clinical Knowledge Summaries (CKS). Gilbert’s syndrome. NICE Clinical Knowledge Summaries (CKS) 2021. Available from: https://cks.nice.org.uk/topics/gilberts-syndrome/. Accessed June 28, 2025.

[4] King D, Armstrong MJ. Overview of Gilbert’s syndrome. *Drug Ther Bull*. 2019;57(2):27–31. doi:10.1136/dtb.2018.000028 30709860

[5] Fretzayas A, Moustaki M, Liapi O, Karpathios T. Gilbert syndrome. *Eur J Pediatr*. 2012;171(1):11–15. doi:10.1007/s00431-011-1641-0 22160004

[6] Kamal S, Abdelhakam S, Ghoraba D, et al. The frequency, clinical course, and health related quality of life in adults with Gilbert’s syndrome: a longitudinal study. *BMC Gastroenterol*. 2019;19(1):22. doi:10.1186/s12876-019-0931-2 30717703 PMC6360704

[7] Preisig D, Bircher J, Preisig R. Positive diagnosis of Gilbert syndrome. Retrospective analysis of 59 cases with special reference to the nicotinic acid test. *Schweiz Med Wochenschr*. 1982;112(33):1122–1129. 7134940

[8] Bale G, Avanthi US, Padaki NR, Sharma M, Duvvur NR, Vishnubhotla VRK. Incidence and risk of gallstone disease in Gilbert’s syndrome patients in Indian population. *J Clin Exp Hepatol*. 2018;8(4):362–366. doi:10.1016/j.jceh.2017.12.006 30563996 PMC6286431

[9] Strassburg CP. Pharmacogenetics of Gilbert’s syndrome. *Pharmacogenomics*. 2008;9(6):703–715. doi:10.2217/14622416.9.6.703 18518849

[10] Claridge LC, Armstrong MJ, Booth C, Gill PS. Gilbert’s syndrome. *BMJ*. 2011;342:d2293. 21508045 10.1136/bmj.d2293

[11] Owens IS, Ritter JK, Yeatman MT, Chen F. The novelUGT1 gene complex links bilirubin, xenobiotics, and therapeutic drug metabolism by encoding UDP-glucuronosyltransferase isozymes with a common carboxyl terminus. *J Pharmacokinet Biopharm*. 1996;24(5):491–508. doi:10.1007/BF02353476 9131487

[12] Yong M, Schwartz SM, Atkinson C, et al. Associations between polymorphisms in glucuronidation and sulfation enzymes and sex steroid concentrations in premenopausal women in the United States. *J Steroid Biochem Mol Biol*. 2011;124(1–2):10–18. doi:10.1016/j.jsbmb.2010.12.014 21193038 PMC3065887

[13] Senan EM, Al-Adhaileh MH, Alsaade FW, et al. Diagnosis of chronic kidney disease using effective classification algorithms and recursive feature elimination techniques. *J Healthc Eng*. 2021;2021:1004767. doi:10.1155/2021/1004767 34211680 PMC8208843

[14] Aggrawal R, Pal S. Multi-machine learning binary classification, feature selection and comparison technique for predicting death events related to heart disease. *Int J Pharm Res*. 2020;13. doi:10.31838/ijpr/2021.13.01.080

[15] Ali RH, Abdulsalam WH. The prediction of COVID 19 disease using feature selection techniques. *J Phys Conf Ser*. 2021;1879(2):022083. doi:10.1088/1742-6596/1879/2/022083

[16] Lis Y. The VAMP research multi-purpose database in the U.K. *J Clin Epidemiol*. 1995;48(3):431–443. doi:10.1016/0895-4356(94)00137-F 7897464

[17] Chisholm J. The read clinical classification. *BMJ*. 1990;300(6732):1092. doi:10.1136/bmj.300.6732.1092 2344534 PMC1662793

[18] Booth N. What are the read codes? *Health Libr Rev*. 1994;11(3):177–182. doi:10.1046/j.1365-2532.1994.1130177.x 10139676

[19] Davé S, Petersen I. Creating medical and drug code lists to identify cases in primary care databases. *Pharmacoepidemiol Drug Saf*. 2009;18(8):704–707. doi:10.1002/pds.1770 19455565

[20] Horsfall LJ, Nazareth I, Pereira SP, Petersen I. Gilbert’s syndrome and the risk of death: a population-based cohort study. *J Gastroenterol Hepatol*. 2013;28(10):1643–1647. doi:10.1111/jgh.12279 23701650

[21] Horsfall L, Walters K, Petersen I. Identifying periods of acceptable computer usage in primary care research databases. *Pharmacoepidemiol Drug Saf*. 2013;22(1):64–69. doi:10.1002/pds.3368 23124958

[22] Blane D, Townsend P, Phillimore P, Beattie A. Health and deprivation: inequality and the north. *Br J Sociol*. 1989;40(2):344. doi:10.2307/590279

[23] Beaumont JJ, Steenland K, Minton A, Meyer S. A computer program for incidence density sampling of controls in case-control studies nested within occupational cohort studies. *Am J Epidemiol*. 1989;129(1):212–219. doi:10.1093/oxfordjournals.aje.a115111 2910063

[24] Stoltzfus JC. Logistic regression: a brief primer. *Acad Emerg Med*. 2011;18(10):1099–1104. doi:10.1111/j.1553-2712.2011.01185.x 21996075

[25] Lewis JD, Bilker WB, Weinstein RB, Strom BL. The relationship between time since registration and measured incidence rates in the general practice research database. *Pharmacoepidemiol Drug Saf*. 2005;14(7):443–451. doi:10.1002/pds.1115 15898131

[26] 8. The chi squared tests. The BMJ | The BMJ: Leading General Medical Journal Research Education Comment 2020. Available from: https://www.bmj.com/about-bmj/resources-readers/publications/statistics-square-one/8-chi-squared-tests. Accessed June 28, 2025.

[27] Tenny S, Boktor SW. *Incidence*. StatPearls Publishing; 2023. 28613497

[28] StataCorp. Stata Statistical Software: release 16. 2020.

[29] van Rossum G. *Python Tutorial, Technical Report CS-R9526*. Amsterdam: Centrum voor Wiskunde en Informatica (CWI);; 1995.

[30] StataCorp. Stata Statistical Software: release 17. 2021.

[31] Powell LW, Hemingway E, Billing BH, Sherlock S. Idiopathic unconjugated hyperbilirubinemia (Gilbert’s syndrome). *N Engl J Med*. 1967;277(21):1108–1112. doi:10.1056/NEJM196711232772102 6054997

[32] Radlović N, Leković Z, Mladenović M, et al. Gilbert’s syndrome in children--our experience. *Srp Arh Celok Lek*. 2007;135(5–6):317–320. doi:10.2298/SARH0706317R 17633320

[33] Sieg A, Arab L, Schlierf G, Stiehl A, Kommerell B. Prevalence of Gilbert’s syndrome in Germany. *Dtsch Med Wochenschr*. 1987;112(31/32):1206–1208. doi:10.1055/s-2008-1068222 3608845

[34] Hemmati F, Saki F, Saki N, Haghighat M. Gilbert syndrome in Iran, Fars Province. *Ann Saudi Med*. 2010;30(1):84. doi:10.5144/0256-4947.59376 20103965 PMC2850189

[35] Méndez L, Lagoa M, Quiroga T, et al. Prevalence of Gilbert syndrome and its genetic determinants in Chile. *Rev Med Chil*. 2013;141(10):1266–1274. doi:10.4067/S0034-98872013001000005 24522354

[36] Gwee KA, Koay ES, Kang JY. The prevalence of isolated unconjugated hyperbilirubinaemia (Gilbert’s syndrome) in subjects attending a health screening programme in Singapore. *Singapore Med J*. 1992;33(6):588–589. 1488666

[37] Kamisako T. What is Gilbert’s syndrome? Lesson from genetic polymorphisms of UGT1A1 in Gilbert’s syndrome from Asia. *J Gastroenterol Hepatol*. 2004;19(9):955–957. doi:10.1111/j.1440-1746.2004.03524.x 15304109

[38] Premawardhena A, Fisher CA, Liu YT, et al. The global distribution of length polymorphisms of the promoters of the glucuronosyltransferase 1 gene (UGT1A1): hematologic and evolutionary implications. *Blood Cells Mol Dis*. 2003;31(1):98–101. doi:10.1016/S1079-9796(03)00071-8 12850492

[39] Wagner K-H, Shiels RG, Lang CA, Seyed Khoei N, Bulmer AC. Diagnostic criteria and contributors to Gilbert’s syndrome. *Crit Rev Clin Lab Sci*. 2018;55(2):129–139. doi:10.1080/10408363.2018.1428526 29390925

[40] Monaghan G, Ryan M, Seddon R, Hume R, Burchell B. Genetic variation in bilirubin UPD-glucuronosyltransferase gene promoter and Gilbert’s syndrome. *Lancet*. 1996;347(9001):578–581. doi:10.1016/S0140-6736(96)91273-8 8596320

[41] Bosma PJ, Chowdhury JR, Bakker C, et al. The genetic basis of the reduced expression of bilirubin UDP-glucuronosyltransferase 1 in Gilbert’s syndrome. *N Engl J Med*. 1995;333(18):1171–1175. doi:10.1056/NEJM199511023331802 7565971

[42] Hamilton FW, Abeysekera KWM, Hamilton W, Timpson NJ. Effect of bilirubin and Gilbert syndrome on health: cohort analysis of observational, genetic, and Mendelian randomisation associations. *BMJ Med*. 2023;2:e000467. 10.1136/bmjmed-2022-000467 PMC10347488 37456363

[43] Vítek L, Tiribelli C. Gilbert’s syndrome revisited. *J Hepatol*. 2023;79(4):1049–1055. doi:10.1016/j.jhep.2023.06.004 37390966

[44] Kang L-L, Ma Y-J, Zhang H-D. Carbon monoxide breath test assessment of mild hemolysis in Gilbert’s syndrome. *Medicine*. 2020;99:e19109. 32049823 10.1097/MD.0000000000019109 PMC7035016

[45] Kaplan M, Hammerman C, Rubaltelli FF, et al. Hemolysis and bilirubin conjugation in association with UDP-glucuronosyltransferase 1A1 promoter polymorphism. *Hepatology*. 2002;35(4):905–911. doi:10.1053/jhep.2002.32526 11915038

[46] Blak BT, Thompson M, Dattani H, Bourke A. Generalisability of The Health Improvement Network (THIN) database: demographics, chronic disease prevalence and mortality rates. *Inform Prim Care*. 2011;19(4):251–255. doi:10.14236/jhi.v19i4.820 22828580

[47] Jani B, Bikker AP, Higgins M, et al. Patient centredness and the outcome of primary care consultations with patients with depression in areas of high and low socioeconomic deprivation. *Br J Gen Pract*. 2012;62(601):e576–81. doi:10.3399/bjgp12X653633 22867682 PMC3404336

[48] Collin SM, Bakken IJ, Nazareth I, Crawley E, White PD. Trends in the incidence of chronic fatigue syndrome and fibromyalgia in the UK, 2001–2013: a clinical practice research datalink study. *J R Soc Med*. 2017;110(6):231–244. doi:10.1177/0141076817702530 28358988 PMC5499564

[49] Hafezparast N, Bragan Turner E, Dunbar-Rees R, et al. Identifying populations with chronic pain in primary care: developing an algorithm and logic rules applied to coded primary care diagnostic and medication data. *BMC Prim Care*. 2023;24(1). doi:10.1186/s12875-023-02134-1 PMC10494405 37691103

